# The dune effect on sand-transporting winds on Mars

**DOI:** 10.1038/ncomms9796

**Published:** 2015-11-05

**Authors:** Derek W. T. Jackson, Mary C Bourke, Thomas A. G. Smyth

**Affiliations:** 1 School of Environmental Sciences, Ulster University, Coleraine BT52 1SA, UK; 2 Department of Geography, Trinity College Dublin, Dublin D2, Ireland; 3 School of the Environment, Earth Sciences Building, Flinders University, GPO Box 2100, Adelaide, South Australia 5001, Australia

## Abstract

Wind on Mars is a significant agent of contemporary surface change, yet the absence of *in situ* meteorological data hampers the understanding of surface–atmospheric interactions. Airflow models at length scales relevant to landform size now enable examination of conditions that might activate even small-scale bedforms (ripples) under certain contemporary wind regimes. Ripples have the potential to be used as modern ‘wind vanes' on Mars. Here we use 3D airflow modelling to demonstrate that local dune topography exerts a strong influence on wind speed and direction and that ripple movement likely reflects steered wind direction for certain dune ridge shapes. The poor correlation of dune orientation with effective sand-transporting winds suggests that large dunes may not be mobile under modelled wind scenarios. This work highlights the need to first model winds at high resolution before inferring regional wind patterns from ripple movement or dune orientations on the surface of Mars today.

## Abstract (web summary)

The absence of *in situ* and long-term meteorological data hampers our understanding of wind movement on Mars. Here, the authors use 3D airflow modelling to investigate small scale ripple migration and suggest that local dune topography exerts a strong influence on wind speed and direction.

## Graphical abstract

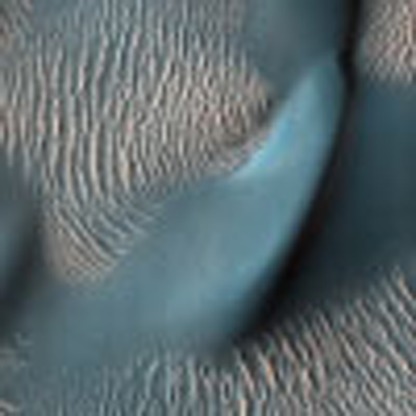

The presence of sand dunes on planetary surfaces can be indicative of past and present wind regimes that have sculpted cohesionless material into organized landforms. These landforms provide a unique insight into the evolutionary dynamics of planetary surfaces and are a window into past climatic behaviour. Reconstructing patterns of regional wind behaviour from the orientation of aeolian bedforms has had limited success due to, for example, model limitations and the, as yet, unstudied role that the inheritance of bedform morphology from previous climatic conditions may play. Unlike dunes on Earth, we still do not fully understand airflow dynamics over and around Martian dunes at relevant scales. If bedforms (for example, dunes or ripple movement on dunes) are to be used as a wind direction proxy, then a better understanding of the controls on ripple migration is needed. Here we use three-dimensional (3D) computational fluid dynamic modelling to demonstrate that wind flow dynamics over and around large-scale dune forms are complex on Mars. Model output in this study was validated using ripple displacements measured from High Resolution Imaging Science Experiment (HiRISE) data. This work advocates the use of detailed, high-resolution surface modelling of winds before attempting to understand regional wind patterns from contemporary bedforms on Mars. In the absence of a network of *in situ* instrumentation to measure winds on Mars, our understanding of airflow over the surface of the planet has relied on large-scale, for example, ref. 1, and meso-scale, for example, ref. 2, 3, atmospheric circulation models along with the interpretation of landform features from satellite images, for example, refs 4, 5, 6. The poor spatial scale of such circulation model data, however, has effectively precluded detailed examination of the forcing mechanisms by which windblown features, such as dunes, move on the surface of Mars. Large- to meso-scale atmospheric circulation models are designed to operate only at scales substantially (2–5 times) larger than the landform feature(s) itself, thereby inhibiting a full understanding of the process response in the system. We must, therefore, adopt a much finer resolution (that is, microscale model) approach to examine the driving mechanisms of any aeolian (windblown) system and its associated landforms. With the availability of high-resolution (0.25 m) HiRISE stereo images of Mars in recent years, high-resolution digital terrain models (DTM) of the surface are now available. This topographic surface can be used to run fine-resolution (sub-landform scale) wind models across complex 3D surface topography such as dune fields.

In the southern hemisphere of Mars, dune fields are contained primarily within large crater basins^7^. The dune sediment is sourced locally from exposed strata in the crater walls and floors^8^,^9^. In some cases (for example, Proctor Crater) the large diameter of these craters means that some intracrater dune fields may be far enough from the crater rim to experience a largely localized wind regime. Here we examine dunes within Proctor Crater, a 150-km diameter impact crater, located within the southern highlands of Mars (47.041°S; 30.667°E) where the dune field is made up of transverse dune forms. This study has shown that detailed 3D modelling of wind on Mars enables us, for the first time, to see the importance of large dune ridge shapes and their complex modification of localized airflow over the surface of Martian dune sites. Results show that back modelling of wind flow from actual ripple movement patterns can identify those regional winds that are forcing the migration of sediment over the dunes on Mars today. We find that winds travelling from the east southeast (ESE) (110°) are the dominant sand-transporting winds, whereas winds from east northeast (ENE) (75°) and west southwest (WSW) (239°) directions appear to play a more subordinate role. Wind modelling on a microscale (5 m) now provides us with an effective new tool to accompany surface ripple displacement information to help understand dune dynamics on Mars.

## Results

### Dune characteristics and wind selection

**Figure 1:**
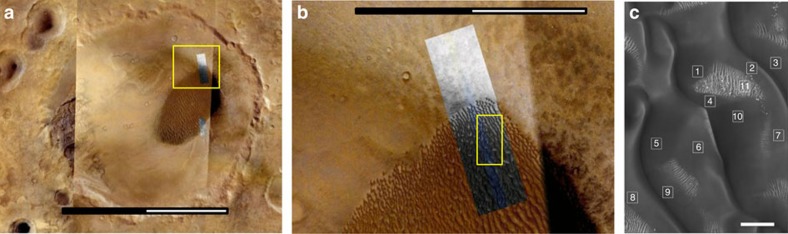
Study location. Regional position of Proctor Crater, area over which airflow model was run and site locations of ripples studied. (**a**) Location of study site within Proctor Crater, (47°41'26.36'S 29°54'53.54E). The dunefield is the dark area in the image. Note: scale bar length is 100 km. The location of our model location is shown in **b**. (**b**) The eastern section of the dune field containing the study area. Note: scale bar length is 40 km (**c**) Image of the surface over which the simulations were run with site locations marked (area demarked in **b**). Note: scale bar length is 400 m.

The dunes at the study site (Fig. 1) are large (500 m wavelength; 70–120 m high) and are aligned transverse to an inferred ENE (75°) incident wind^10^. Bright dune forms are located on the floor of the crater and are in the interdune areas. These have a wavelength of 23 m and are considered immobile under current conditions and are most likely transverse granule ripples^10^.

A 1 m resolution DTM (Fig. 1b) was used for the 3D computational fluid dynamics (CFD) modelling^11^. We selected three dominant wind directions to model; 239° WSW (primary), 110° ESE (secondary) and 75° ENE (tertiary). These winds were identified in earlier work^10^ on Proctor Crater as representing the dominant wind regimes indicated from dune slip-face dip orientations. HiRISE image data that were acquired two Mars years apart were used to measure ripple displacement at 11 sites (Fig. 1c) on three of the largest dunes. Each sample quadrant measured ripple displacement distance and direction over a 10,000 m^2^ area.

### 3D airflow modelling

**Figure 2:**
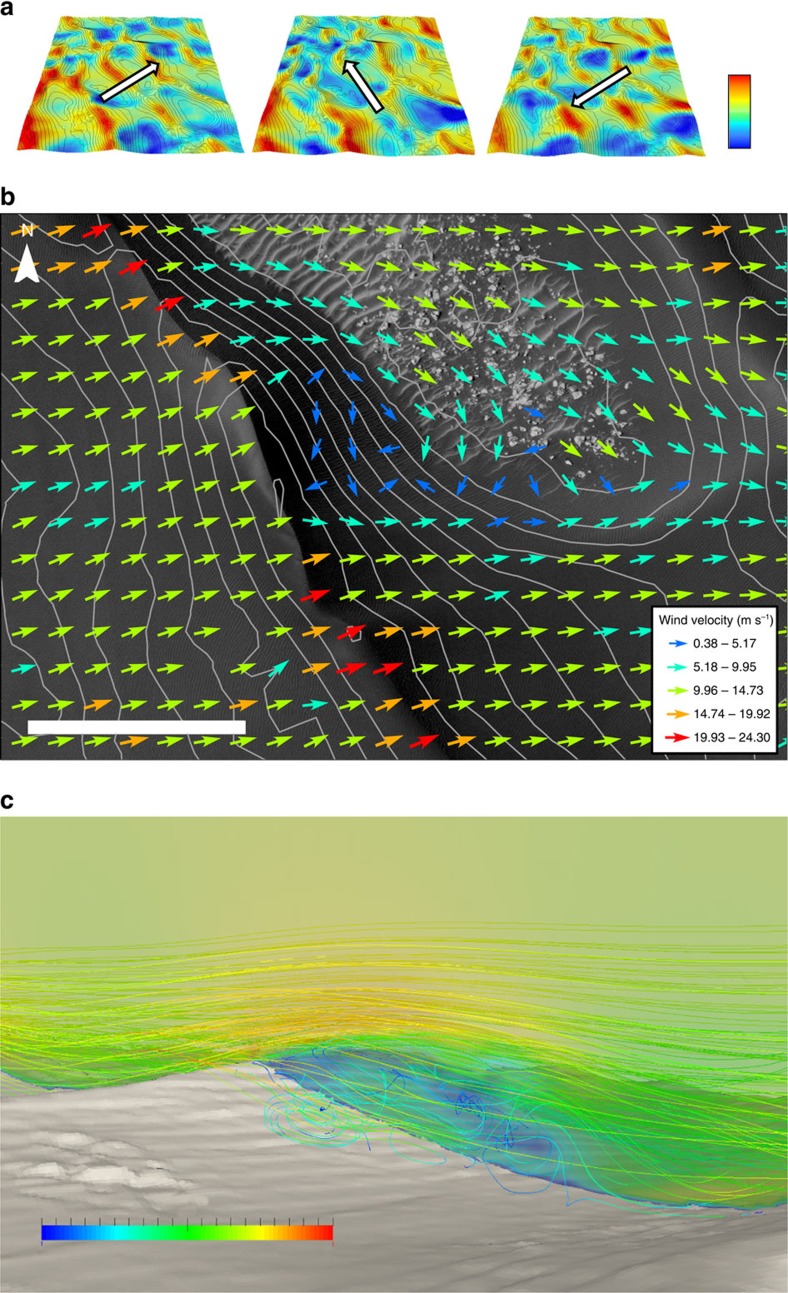
Airflow modelling results near the dune field surface from three simulated directions. (**a**) Primary, secondary and tertiary wind flow at 3 m above the surface over the entire dune field surface identified in Fig. 1b. Note: scale bar range is 0 m s^−1^ (blue) to 21 m s^−1^ (red). These wind directions were adopted from Fenton *et al.*^8^ who used the slip-face dip azimuths of transverse ridges to derive the primary secondary and tertiary directions. The arrow in the centre of each image represents the incident wind direction at the boundary of the computational domain. Data show topographically accelerated wind velocities at dune crests with maximum wind speeds of 24.3 m s^−1^ and ‘dead zones' (velocities ≤1 m s^−1^) in the interdunes for all three modelled winds. Contour data at 10-m interval derived from a DTM was built using HiRISE stereo image pairs PSP_003800_1325 and PSP_004077_1325. The DTM data were made available by NASA/JPL/University of Arizona. The region shown in **a** is the same as that delineated in Fig. 1c. (**b**) Flow steering by a transverse dune (100 m high) under primary incident wind conditions (239°) south of site 8 (fig. 1c). Vectors indicate wind flow at 3 m above the surface, spaced at 50-m intervals. Contours are plotted at 10-m intervals. Note: scale bar range is 300 m. (**c**) A 3D view of the detached flow for primary winds (239°) at site 8 (location in fig. 1c). The perspective of the image is towards the north-west. Incident wind flow (m s^−1^) is moving from left to right in the image. Site 8 is situated immediately north of the frame in **c**. Note: scale bar range is 0 m s^−1^ (blue) to 20 m s^−1^ (red).

Results from CFD modelling allow us to examine the detailed wind flow behaviour and its corresponding association with underlying dune topography at the site within Proctor Crater. High-resolution-modelled winds are seen to vary extensively across the complex dune topography (Fig. 2a), with a general picture of maximum wind (acceleration) along the crestal brinks and minima in the dune troughs. Flows accelerate (relative to incident flow) by up to 150–200% at crest positions, displaying a similar behaviour to dune sites on Earth^12^,^13^. Deceleration of flow is evident in dune trough locations, which coincides with the brighter dune forms in these locations that show no evidence of recent movement in our data (site 11).

Distinctive steering of flow (Fig. 2b) results from pre-existing dune topography. The large dunes differentially retard the flow from oblique directions, forcing the flow into orientations significantly offset from the upwind direction. The specific crestline orientation relative to the upwind flow direction is of primary importance to the degree of steering.

Overall, steered wind directions mapped over the ripple study sites are an average of only 8 degrees offset from the average ripple migration direction. This demonstrates excellent agreement with CFD-modelled flow and localized surface response.

### Ripple movement and modelled localized wind patterns

As the crestal positions of the smaller scale ripples (wavelength 3.4 m) in this study have been shown to move on the order of 0.5–2.0 m over a two Mars–year period, they are considered to be a true response to the modern winds we see on Mars today. Our data show that five of the ripple displacement study sites (2, 4, 6, 8 and 9) have very good correspondence with secondary winds (110°) that have undergone localized steering, displaying only 1–12 degrees of difference between ripple migration direction and steered wind direction. Note that for sites 7, 10 and 11 migration was below detection limits or showed no motion. Study sites 1 and 3 map better onto steered tertiary (75°) incident winds (3 and 19 degree differences, respectfully), while only site 5 mapped onto steered primary (239°) winds.

Our data show decreased flow steering at rounded crests (bottom of Fig. 2b) relative to sharper crests (middle of Fig. 2b). Under circumstances where flow steering has not taken place, the cross-sectional topography dictates if the flow becomes detached, forming eddies that shear off the crestal areas (Fig. 2c). Both flow steering and detachment behaviour modelled for Mars is similar to Earth where crestal sharpness and surface undulations exert a major control^14^,^15^.

## Discussion

**Figure 3:**
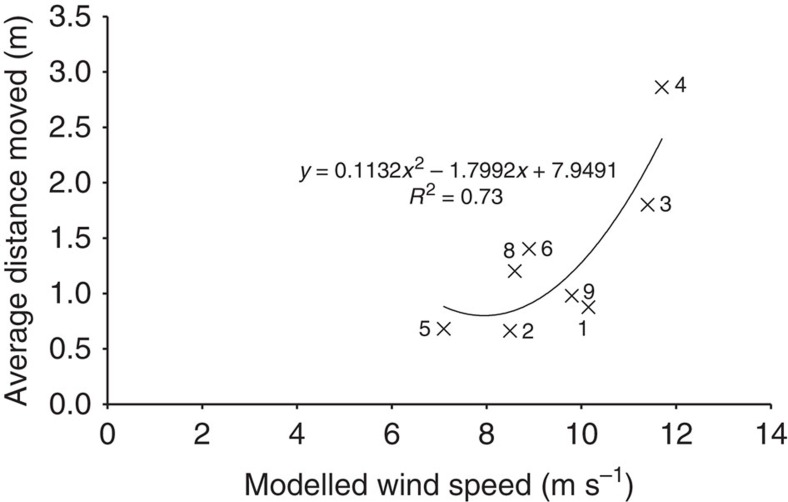
Ripple migration distance versus locally modelled wind velocity. Relationship between the average observed ripple migration distance over 3.8 Earth years (2.0 Mars years) and local CFD wind velocity.

In terms of the modern surface response to wind forcing, an examination of ripple movement provides some intriguing insights into the dominant incident wind directions that are responsible for active migration of ripple sets^16^. For the ripple sites investigated, we find that all three incident wind directions move ripples and, in general, the use of ripples as a tool for back modelling regional winds can be strengthened from the results presented here. However, the large dune topography exerts a significant control on steering localized winds to produce the ripple migration directions. This has a direct control on the mobility of surface sediments and dictates the fate of surface behaviour, contributing to actively migrating surface ripples. Therefore, the ripple direction may reflect a topographically steered wind direction rather than the regional wind regime. Interestingly, when we plot steered wind speed at ripple sites against observed ripple average migration rates (Fig. 3), we see a good correlation (*r*^2^, 0.7), further substantiating that CFD-modelled results are concurrent with ripple response displaying an exponential increase in migration distance with velocity increase.

Modelling in 3D enables us for the first time to see the importance that large dune ridge shape plays on modifying localized flow to give either steered or detached behaviour. We would expect to see a similar localization of winds with strong topographically steered and altered winds for comparable dune topography and wind regimes of similar magnitude and directions elsewhere on Mars.

The physical cross-sectional dimensions of the main dune ridges appear to play an important role in deflecting and steering the localized flow, and seem to dictate which incident flow drives particular ripple set migration directions. Sharply peaked dune crests are likely to steer winds and may also lead to detached flow and large-scale return eddies moving in the opposite direction to incident flow. Rounded dune topographies are less likely to induce this behaviour of detachment and flow maintains itself largely in the same direction as the incident winds albeit steered somewhat in certain cases.

Back modelling–wind flow orthogonally from ripple migration directions examined here, identifies the regional winds that are driving the migration of sediment. Our data show that in the Proctor Crater region, secondary wind travelling from the ESE (110°) is the dominant sand-transporting wind in the dune field. Winds from the tertiary ENE (75°) and primary WSW (239°) directions appear to play a more subordinate role at this site.

The dune slip-face orientations suggest that the formative dune winds are from the ENE^10^ (that is, the tertiary winds). The low correlation of tertiary wind direction with active sediment movement in ripples at the Proctor Crater site suggests that the large dune morphology we studied may not be currently maintained by tertiary winds. Rather, the geomorphologically effective winds are from the ESE (that is, the secondary winds). This opens the possibility that the largest dunes at our site in Proctor Crater may be emplaced during former climate conditions.

Other studies have used the brink orientation on large dunes to infer modern regional-scale wind direction. We advocate caution in using this approach and suggest that further study is required before we can confidently infer modern regional wind direction from the brink orientations of large dunes on Mars. Our findings, along with model limitations, may go some way towards explaining the poor correlation between meso-scale model output and mapped inter-crater dune orientations, for example, ref. 17.

Our microscale wind model provides a new tool whereby mapped ripple displacement directions can be used to back-model the incident and formative wind at timescales relevant to modern Martian aeolian dynamics.

## Methods

### Ripple mapping

For the time-series analysis we used two HiRISE images (PSP_003800_1325 and ESP_021469_1325) that were acquired 3.8 Earth years apart. The images were selected for minimal differences in the orbital parameter and emission angle (3.4°) that reduces parallax distortions. Radiometric distortions are also minimal. The images were acquired at similar times in the season (*L*_s_=240.9° and 242.7°) and at a similar time of day, minimizing radiometric distortions due to different illumination conditions and effects of potential shadow length.

Eleven sites were selected for ripple analysis and were located on dune flanks and in the interdune of three consecutive transverse ridges. The transverse ridges are spaced ∼500 m apart and attain heights of 100 m. Ripple locations were mapped on the two georectified images in ARCGIS and displacement directions and distances were measured.

### Wind flow modelling

Near-surface airflow over the study area was simulated using OpenFOAM, a CFD toolbox that integrates the principal equations of fluid of flow over a domain by converting the integral equations to algebraic equations, before solving them iteratively^18^. Flow was calculated using Reynolds-averaged navier-stokes equations, which average the motion of fluid flow over time. Turbulence was modelled using the two-equation re-normalized group *k*-ɛ turbulence model. The mesh domain measured 2,900 × 3,535 × 600 m and contained 3.8 million cells. Cell size gradually decreased from the top (maximum size 20 × 20 m ) of the computational domain towards the surface to a minimum resolution of 5 × 5 × 2.5 m.

In terms of the inlet static stability profile at the site, temperature was not calculated in each simulation and, therefore, the vertical wind profile is assumed static at the inlet with no consideration made of diurnal temperature change. Detailed evaluation of patterns of diurnal variability was not the focus of this study and instead topographically induced impacts as a result of local dune topography were highlighted. In each case, wind was defined at the inlet boundary as logarithmic using Richards and Hoxey^19^ equations for *k*-ɛ turbulence models (equation 1).


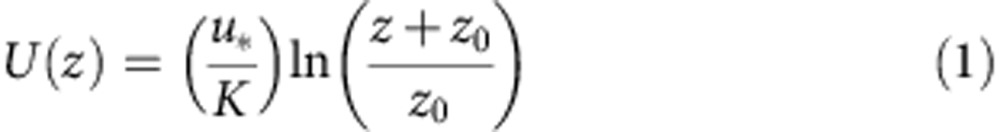


Where *U*(*z*) is the wind speed at height *z*, 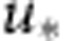 is shear velocity, *K* is the Von Karman constant (0.4187) and *z*_0_ is the aerodynamic roughness. Note that *z*_0_ was attributed a value of 0.05 m, typical of a semi-arid sparse brush^20^ or sparse grass 0.5 m high^21^. With larger roughness lengths of 0.06–0.07 m, near-surface airflow speeds would have been only marginally lower and zones of separation/recirculation marginally greater. These results on steep dunes are, however, negligible^22^. 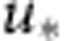
was prescribed a value of 1.45 m s^−1^, producing a wind speed of 20 m s^−1^ at 30 m above the surface at the inlet. Turbulence kinetic energy (*k* ) and energy dissipation (ɛ) were simulated at the inlet boundary using conditions prescribed by Richards and Hoxey^19^ equations 2 and 3.


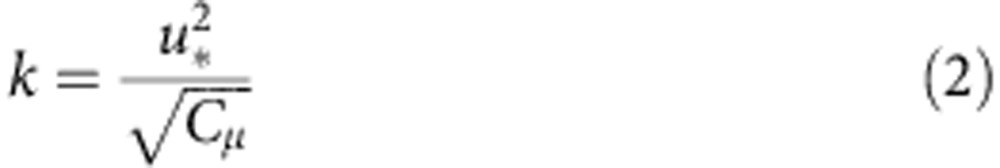



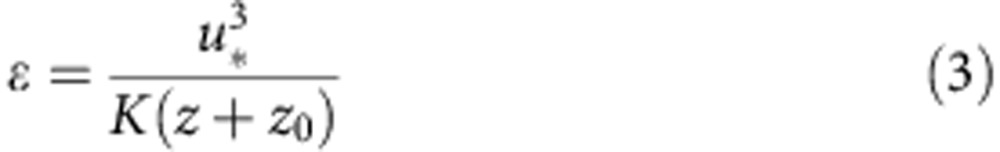


Where *C*_*μ*_ is a constant of the *k*-ɛ model and equals 0.09. The atmospheric kinematic viscosity was specified as 0.0011, m^2^ s^−1^, this value assumes that the atmosphere is composed of 100% CO_2_ at a temperature of 5 °C. Each simulation was considered complete and converged when wind speed, which was probed at 16 equally spaced points within each of the ripple sites, became steady to the third decimal place.

## Additional information

**How to cite this article:** Jackson, D. W. T. *et al.* The dune effect on sand-transporting winds on Mars. *Nat. Commun.* 6:8796 doi: 10.1038/ncomms9796 (2015).
